# Effect of drought stress during critical developmental stages on morphological and grain yield-related traits in winter barley (*Hordeum vulgare* L.)

**DOI:** 10.1371/journal.pone.0329391

**Published:** 2025-07-30

**Authors:** Zita Berki, Tibor Kiss, Judit Bányai, András Cseh, Krisztina Balla, Ildikó Karsai

**Affiliations:** 1 HUN-REN Centre for Agricultural Research, Agricultural Institute, Martonvásár, Hungary; 2 Food and Wine Research Institute, Eszterházy Károly Catholic University, Eger, Hungary; ICAR Indian Institute of Wheat and Barley Research, INDIA

## Abstract

As the frequency of droughts increases, the breeding of new drought-tolerant cereal varieties may become increasingly important. However, the complex effects of drought stress on grain yield-related traits are difficult to study precisely, and relatively little information is available on how drought during flowering affects plants. Therefore, 28 winter barley cultivars were included into controlled environmental tests, where their reactions were determined to single drought stress treatment applied at heading and to combined drought stresses applied at first node appearance and then at heading. Drought stress (both single and combined) significantly reduced all of the grain-yield related traits. Notably, grain yield was reduced by 48% in the two-row varieties and by 44.24% in the six-row varieties under combined drought stress, compared to the control. Our study has also demonstrated, that the combined application of drought tolerance/susceptibility indices (DT/SIs) and BLUP-based analysis provides a reliable approach for identifying stress-tolerant genotypes. We identified two main types of drought stress tolerance: the ability of preserving grain number and weight in the main ears, in parallel of maintaining the number of reproductive tillers (more tolerant), and the ability of preserving grain number and weight in the side ears (least tolerant). Both types appeared in either treatment, but not with the same intensity. Our results may provide useful information for a better understanding of this topic, which may become even more important in the context of increasingly frequent droughts.

## Introduction

According to the IPCC’s Sixth Assessment Report [1] the appearance of the extreme weather elements in the future will be more frequent as the consequence of the changing climate. Plants are known as sessile organisms, exposed to various environmental stresses during their growing season. Of these, drought is one of the most important environmental stress parameters and the plant response to it is a complex phenomenon (regulated by many genes), therefore to maintain the yield stability in drought-prone areas, it requires the collections of in-depth and extensive knowledge in order to be able to produce newer varieties that are better adapted to the changing environmental conditions [2]. Various molecular-genetic, biochemical, physiological and morphological traits and processes of plants can also be affected by heat and drought stress [3,4], leading to a reduction of the yield in the crop production worldwide by 50% or more [4–8]. Thus, the grain yield in different environments (especially in arid and semi-arid areas) should be used as the main indicator of drought tolerance [9–12]. Selection of more tolerant varieties based on only the grain yield is, however difficult, due to the low heritability usually registered under drought conditions, as a consequence of the high level of variance in genotype × environment interactions, and to the low variation in the genotypic responses under stressful conditions [13–17]. Therefore, alongside the selection for grain-yield related yield components, it is essential to consider drought tolerance and susceptibility indices (DT/SIs) in the breeding of drought-tolerant varieties [18,19]. These include, for example, the Drought Susceptibility Index (DSI; [20], where DSI < 1: more tolerant to drought, while DSI > 1: more sensitive to drought [21]), the Stress Tolerance Index (STI; [22], where STI ≥ 1: high tolerant, 0.5 ≤ STI < 1: moderately tolerant, STI < 0.5: low tolerance [23]), the Yield Stability Index (YSI; [24], where higher-valued genotypes are more stable under stress conditions, while low-valued genotypes are less stable under stress conditions [25]), and the Tolerance Index (TOL; [26], where low-valued genotypes are more stable under stress conditions, while higher-valued genotypes are less stable under stress conditions [25]). Specifically, DSI, STI, YSI, and TOL have been identified as useful indicators of yield-related traits in crops under different water regimes [27–29]. Furthermore, the Best Linear Unbiased Prediction (BLUP) method can also be used to analyse the effectiveness of selection indicators [30]. These parametric stability measures have been widely applied and reported for assessing the degree of genotype-by-environment interaction (GEI) in barley [28,31–37]. Drought stress responses are regulated by intricate physiological, biochemical, and molecular-genetic mechanisms, and recommending the appropriate index for selecting tolerant genotypes is indeed a difficult and complex challenge [28,38]. Consequently, no single index can effectively identify high-yielding genotypes under stress conditions. Furthermore, the effectiveness of these indices in screening also depends on the severity of the stress [39].

Throughout evolution, plants have evolved different adaptive mechanisms to cope with the adverse effects of drought stress. Stress avoidance, escape and tolerance are the most important survival strategies [4,40]. To develop new drought tolerant crops, not only traditional breeding methods but also molecular genetic techniques are increasingly being used [41]. A better understanding of the genetic components of the different regulatory cascades may enhance the ability to manipulate the adaptive capacity of different crops and thereby their productivity [4,42,43]. Therefore, it is important to examine the consequences of water deficit on the economically important cereal crops, including barley [44,45]. In general, barley shows high genetic variability in abiotic stress tolerance responses (grown in a wider range of environmental conditions), which makes it an excellent model crop for studying the genetic regulation of adaptation to water deficiency [2,46,47]. In addition, the relatively simple diploid genome of barley and its close relationship to other cereals in the grass family of Poaceae (e.g., *Triticeae*) may facilitate the use of the knowledge gained here for drought stress tolerance studies in wheat and rye, as well. Therefore, barley is a promising gene source for drought research.

Increase in the appearance and intensity of drought periods highly affect various aspects of plant development in barley [48]. Certain developmental stages have been identified that are critically sensitive to the water distraction, such as first node appearance, flowering [3,49] or grain filling [50–55]. Drought stress during these stages affects many morphological and yield components: plant height, number of spikes, number of kernels per spike, grain/kernel weight per spike, number of fertile florets or grains per m^2^, number of tillers, photosynthetic rate and harvest index [9,53,56]. Drought mostly impairs spike development, and is therefore a major cause of yield losses in the temperate cereals [57]. Less spikes are formed on the tillers, accompanied by reduced spikelet and fertile floret numbers [54,58–63]. The consequences of drought stress appear not only in the yield loss, but also in the reduced quality of the seeds [3,64,65]. In addition, a common phenomenon is that the grains in the basal and apical spikelets suffer disproportionately greater size and weight loss, due to the disturbance of the grain filling phase [66–68].

Drought stress can be examined from more aspects. Some experiments are highlighted the levels of drought stress [69,70], and also there are those that investigate the water deficit in different growth stages [70]. Most studies of drought stress focus on water subtraction in single developmental stage [71–73], or they combine drought stress with heat stress [74–76], but there is not much information about the consequences of water deficit in combination of early and late developmental stages [48]. Therefore, the main aim of our study was to compare the reaction of different barley cultivars (28) to a single and to a combined drought stress treatment in controlled environmental conditions (phytotron) on the morphological and yield components examined. In addition to the yield component related analyses, four drought tolerance/susceptibility indices (DSI, STI, YSI and TOL) were also evaluated, and BLUP analysis was used to confirm the genotypes of the stress-tolerant and susceptible groups. Determining the main stress tolerance type among 28 barley genotypes was carried out by applying hierarchical cluster analysis. We also hypothesized, that cumulative drought stress at both early and late developmental stages would lead to greater stress tolerance compared to single stress, due to a possible stress priming effect of epigenetic modifications [77]. The same group of barley cultivars were included in the drought stress experiments, whose response to heat stress has already been published by Horváth [77]. Single drought stress was used at heading, while combined drought stress at first node appearance followed by an additional stress at heading stage. The comparison of the response of drought resistant genotypes could contribute to the breeding of new varieties with higher drought tolerance level. Based on the changes due to the predicted global warming, the duration and severity of the dry periods may occur more frequently during any of the different plant developmental stages (intensive stem elongation, heading and grain filling) and unpredictably every year. Thus, the consequences of the changing climate on the different cereal species needs to be clarified and not only in the terminal developmental stages.

## Materials and methods

### Plant material

190 winter and facultative barley cultivars were selected to form a panel for examining barley ecological adaptation, yield formation and abiotic stress tolerance in the Centre for Agricultural Research (HUN-REN ATK), Martonvásár, Hungary [77]. In the framework of our research, this panel was genotyped with the 45K Infinium SNP chip of TraitGenetics and phenotyped in a field-sown experiment in 2018 [77]. From this panel a subset of 28 winter barley cultivars of various geographic origins were included in the controlled drought stress experiment (S1 Table). They were the same ones that were studied for heat stress tolerance by Horváth [77]. Additional information on the origin, on the selection process and on their genetic diversity can be found in [77,78].

### Conditions in drought stress experiment

The experiment was carried out in the Phytotron facilities of HUN-REN ATK, Martonvásár using CONVIRON PGB-96 growth chambers (Conviron, Winnipeg, MB, Canada). The environmental conditions were constant throughout the experiment: a 16-h photoperiod, a PAR light intensity of 240 µmolm^-2^s^-1^ provided by metal halide lamps, and a constant ambient temperature of 18°C day and night. The experiment consisted of three treatments: control (C), single drought stress applied at the booting stage (Z49, [79]. referred to as Ds), and combined drought stress (double) applied at first node appearance and after a recovery period again at booting stage (Z31 + Z49; referred to as Dd). For all three treatments, the germinated seedlings were vernalized for 60 days in Jiffy^®^ pots (Zwijndrecht, Netherlands) at 4˚C, low light intensity (24 µmolm-2s-1 of PAR) and short days (9-h photoperiod). The vernalized plantlets of one-two leaf stages were then transferred to individual pots (16 cm × 16 cm × 16 cm) containing a 3:2:1 mixture of about 2.5 kg of garden soil, compost and sand. For each barley genotype and each treatment, 24 plants were planted and 4 plants were grown in individual pots. During the stress treatments, the watering of the plants was stopped till the soil moisture content dropped to 15 vol% [48], this level was kept for 7 days then re-watering was applied. In the control treatment a constant soil moisture content was maintained on 27 vol%. Three sensors (5TE- Decagon Devices, USA) were placed in every treatment. Soil moisture (tf%), soil temperature (°C) and electrical conductivity (mS/cm) were recorded in every two hours, from planting till maturity.

The 24 replicates (plants) of each treatment and each genotype were harvested at full maturity and the following morphological and yield related parameters were measured: length of the last internode (LIN), length of the main ear (EaL), number of spikelets in the main ear (SPS) main ear density (DENS), number of reproductive tillers (RT), aboveground dry weight of plant without the ears (BIOM), grain number per spikelet of the main ear (MSSN), main ear weight (MEaW), weight of grains in the main ear (MSW), number of grains in the main ear (MSN), total side ears weight (SEaW), total grain weight and grain number in the side ears (SSW and SSN), and grain yield per plant (GY). Thousand kernel weight in the main ear (MTKW), average thousand kernel weight (ATKW), average number and weight of grains per spikes harvested (ASN and ASW) were also calculated from these data.

### Statistical analysis

In the statistical analysis, the average of 4 plants per pot was considered as 1 replicate, so 4 replicates per genotype (4 × 4 plants) were used in the analyses (the two outer pots were not considered), in both the control and the two drought stress treatments. These data are summarised in the S2 Table.

The general statistical analyses were carried out by the software packages of RStudio 4.1.1 [80] and GeneStat [81]. The *tidyverse* package was used to generate general descriptive statistics (mean, standard deviation, standard error) [82]. Heatmaps and hierarchical cluster analyses were conducted using the *heatmaply* and *dendextend* packages, based on the percentage values of treatments compared to the control, in order to be able to compare the effect of the two stress treatments in all genotypes [83,84]. Using the gg*corrplot* package, we visualized complex relationships between variables in large datasets for our analyses using Pearson’s correlation statistics [85]. Boxplots was employed for visualizing the results about the distributions of the original data under the various treatments by the R package *ggplot2* [86]. In the three different environments (control, single and double drought stress), the networks between all the examined morphological and yield related traits were visualized with R qgraph 1.9.2. [87]. Principal component analysis (PCA) and its representation in biplot image were carried out by the R packages *Factoextra* [88,89] and *FactoMineR* [90]. The two-way ANOVA analysis was done by IBM SPSS Statistics [91]. All DT/SI indices and BLUP-based analysis were calculated based on GY to determine tolerant genotypes with high yield potential averaged across the two stress treatments (single and double drought stress).

The Drought Susceptibility Index (DSI) was calculated using the following formula [20],


$$\begin{array}{c} \text{DSI}\;=\;\left[1\;-\;\text{Ys/\textbackslash{};Yp}\right]\text{/SI} \end{array}$$

Stress Intensity (SI) was calculated using the equation below [20],


$$\text{SI}\;=\;1\;-\;\left(\overset{―}{\text{Y}}\text{s}/\;\overset{―}{\text{Y}}\text{p}\right)$$

The Stress Tolerance Index (STI) was calculated using the following formula [22],


$$\text{STI}\;=\;\left(\text{Ys}\;\times \;\text{Yp}\right)/\left(\overset{―}{\text{Y}}\text{p}\right)2$$

The Yield Stability Index (YSI) was calculated using the following formula [24],


YSI=Ys/Yp

The Tolerance Index (TOL) was calculated using the following formula [26],


TOL=Yp\;-\;Ys

where, Ys and Yp are trait (grain yield – GY was used) mean for each genotype in drought stress and control conditions, respectively. Furthermore, Ȳs and Ȳp are grand means for grain yield (GY) in drought stress and control conditions, respectively. Drought stress was defined as the mean of the values from the two treatments (single and double), as in general no significant difference was found between them. The BLUP was applied within the framework of the linear model described by Olivoto [92]. This method was used to confirm the grouping of genotypes into drought stress-tolerant and susceptible categories. In BLUP, genotype was assumed as fix, and traits and treatments as random effects. The normality assumption which is required for BLUP was graphically checked in Q-Q plots [30]. The BLUP values were estimated using R package *metan* [93].

## Results

### Effect of drought stress on grain yield related traits in barley

In this study the yield components were used to investigate the stress tolerance of the 28 barley cultivars. Drought stress was applied in a developmental stage specific manner due to the nature of controlled environments. Single drought stress (Ds) was applied at the heading stage of each genotype (Z49), while the double drought stress (Dd) comprised of first stress treatment at the first node appearance (Z31), and of a second stress treatment at the heading stage (Z49). Based on the two-way ANOVA, both drought treatment and genotype were significant factors in determining the various parameters, but their ratio varied strongly with the individual traits (S1 Fig). To exclude the differences caused by the head row types, the results were separately visualized in the two groups of two rowed and six rowed varieties. Both morphological and yield components, were mainly determined by treatment, independently from row-type (46.6–97.1%). However, for number of reproductive tillers (RT), ear density of the main ear (Dens), above ground biomass (BIOM), grain number in one spikelet of the main ear (MSSN) and total grain numbers in the side ears (SSN), the genotype exerted a greater effect (40–73%) (S1 Fig).

**Fig 1:**
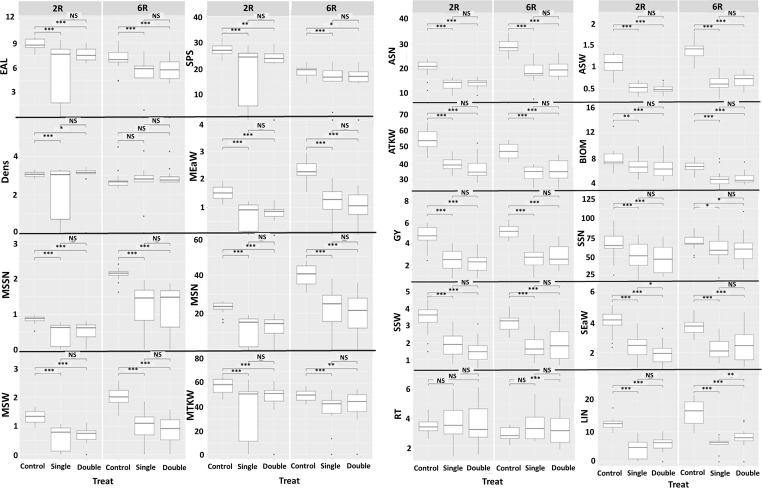
Influences of single and combined drought stress on the five morphological and thirteen yield-related traits investigated on group averages of 28 barley cultivars. (2R = two-rowed, 6R = six-rowed). Yield-related traits: **ASN** = Average number of grains per spike harvested, **ASW** = Average weight of grains per spikes harvested, **ATKW** = Averages thousand kernel weight, **GY** = Grain yield per plant, **MEaW** = Main ear weight, **MSN** = Number of grains in the main ear, **MSSN** = Seed number per spikelet of the main ear, **MSW** = Weight of grains in the main ear, **MTKW** = Thousand kernel weight in the main ear **RT** = Number of the reproductive tillers, **SEaW** = Total side ears weight, **SSN** = Total grain number in side ears, **SSW** = Total grain weight in the side ears. Morphological traits: **BIOM** = Aboveground dry weight of plant without the ears, **Dens** = Main ear density, **EaL** = Length of the main ear, **LIN** = Length of the last internode, **SPS** = number of spikelets in the main ear. *, **, *** denote significant relationships at the P ≤ 0.05, P ≤ 0.01 and P ≤ 0.001 probability levels, respectively; ns (not significant).

Most traits were negatively affected to varying degrees by the drought stress treatments. Since the barley row-type basically determines several parameters related to grain yield, the means and intervals of the row types are shown separately in Fig 1.

For most traits, combined stress had similar effects to single stress, with little or no difference between them. For the control, the median MSN for six-row barley was 40.63 grains, with a wider range varying by treatment and variety. After the single treatment, MSN decreased to 25.13 grains, while after the combined treatment to 20.5. These values for the two-row type were 24, 14.13 and 14.25, respectively. Regardless of the row-type, MSW was also significantly negatively affected by single and combined drought stress. Overall, MSW showed a 53.12 and 54.42% reduction in response to single and combined treatments, respectively, compared to the control, regardless of row-type. For MTKW, this pair of ratios was 32.08 and 20.62%. GY of both two-row and six-row barley varieties were strongly affected by the exposure to drought stress. In the two-row varieties, the average yield in the control treatment was 4.7 grams, which decreased by 48% under the stress, resulting in a mean yield of 2.45 grams in the single drought stress. This further decreased to 2.2 grams in the combined treatment. For the six-row varieties, a yield reduction of 47.52% was observed after the single treatment, whereas after the combined treatment the plants performed on average 44.24% below the control. Similar tendencies were also observed for average number of grains, weight and thousand kernel weight of side tillers.

### Genotypic differences in responses to single drought stress

**Fig 2:**
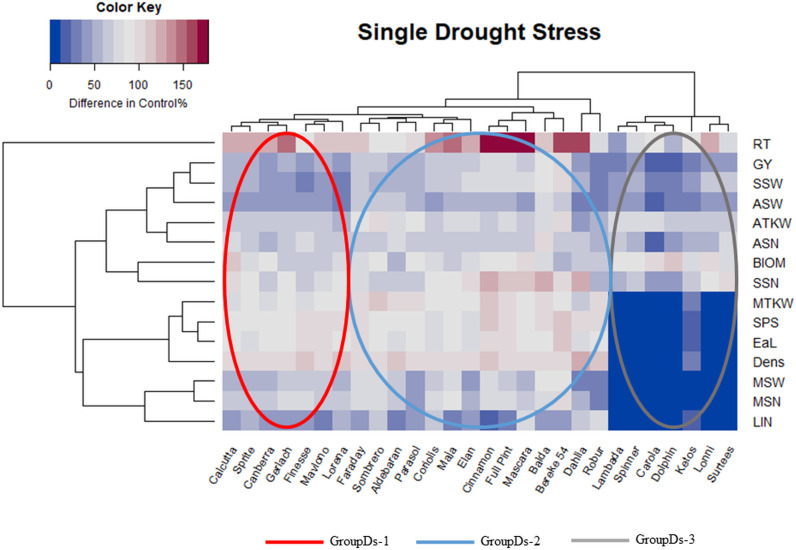
Heatmap combined with dendrograms of 28 barley genotypes (columns) and 15 traits (rows) using a single drought stress applied at the booting stage (Z49), expressed through changes in the percentage of the control values. The statistical methods used and the results visualized were the same as the ones used by our research group before [77]. **Abbreviations**: RT – number of reproductive tillers, GY – grain yield per plant, SSW – total grain weight in the side ears, ASW – average weight of grains per spikes harvested, ATKW – average thousand kernel weight, ASN – average number of grains per spikes harvested, BIOM* – aboveground dry weight of plant without the ears, SSN – total grain number in side ears, MTKW – thousand kernel weight in the main ear, SPS* – number of spikelets in the main ear, EaL* – length of the main ear, DENS* – main ear density, MSW – weight of the grains in the main ear, MSN – number of grains in the main ear, LIN* – length of last internode. *morphological traits.

In order to evaluate the individual genotype reactions in more details and to balance the deviations in units and magnitudes of traits, a heatmap construction was performed in such a way that for each variety the measured traits were expressed as a % of control treatment. This matrix was used to prepare the heatmap, based on which simultaneous grouping of the traits and genotypes became possible based on their reaction to drought stress (Fig 2).

**Fig 3:**
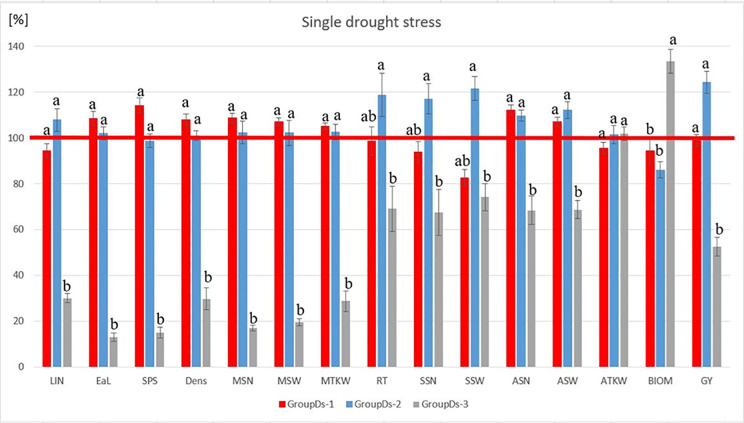
Evaluation of the stress responses of the three barley groups (GroupDs), identified in the heatmap using a single drought stress treatment applied at booting stage (Z49). (The calculation of group averages is based on the individual genotypic change percentage values, expressed as a proportion of the overall average change percentage of the 28 genotypes. The bars represent the standard deviation of the group members for each parameter. The same letters represented are not significantly different from one another at the P = 0.05 level). The statistical methods used and the results visualized were the same as the ones used by our research group before [77]. **Abbreviations**: LIN* – length of last internode, EaL* – length of the main ear, SPS* – number of spikelets in the main ear, DENS* – main ear density, MSN – number of grains in the main ear, MSW – weight of the grains in the main ear, MTKW – thousand kernel weight in the main ear, RT – number of reproductive tillers, SSN – total grain number in side ears, SSW – total grain weight in the side ears, ASN – average number of grains per spikes harvested, ASW – average weight of grains per spikes harvested, ATKW – average thousand kernel weight, BIOM* – aboveground dry weight of plant without the ears, GY – grain yield per plant. *morphological traits.

After single drought stress, four subclusters of the traits were formed. A completely separate subcluster contained only the number of reproductive tillers (RT) which showed mostly increased values. In a subcluster, GY correlated with the total grain weight in the side ears (SSW) and the average weight of grains per spikes (ASW) to the largest extent. The grain numbers (ASN and SSN), the amount of biomass (BIOM) and the average thousand kernel weight (ATKW) formed the next subcluster. Together with the fourth subcluster including ear parameters (MSN, MSW and MTKW) and the morphological traits (EaL, SPS, DENS and LIN), they were more distinctly separated from that including GY. The associations of the reproductive tiller number were weak with all of the other parameters. In analysing the groupings of the barley genotypes, it became evident, that the different grain characteristics decreased to various extents compared to the control values in a genotypic manner. Based on the range of negative to positive values in the morphological and yield related traits, the 28 barley genotypes formed three distinct groups. All three groups contained both two and six row barleys. The most drought sensitive genotypes formed Group 3, while barleys with better tolerances belonged to Group 1 and 2. In the following analysis, the groups are referred to as GroupDs. In order to be able to better discriminate the basis of stress responses of the three genotype groups, the means of the three groups were compared (Fig 3).

Under control treatment, the group means did not differ significantly from each other with the exceptions of ATKW and BIOM. Under single drought stress, however, there was larger difference between the three groups. GY of the three GroupDs remained well below the grand mean of change% of the 28 barleys, (51.7, 64.99 and 27.42%, respectively). The basis of better stress tolerance levels detected in GroupDs-2 and to a smaller extent in GroupDs-1 were considerably distinct from GroupDs-3. Genotypes in GroupDs-1 and GroupDs-2 were better able to maintain the grain number and weight in the main ears (MSN, MSW and MTKW) during the drought stress period. In addition to the almost similar number of reproductive tillers, genotypes in GroupDs-1 and GroupDs-2 were able to produce a larger total grain number (SSN) and weights (SSW) under the recovery period. Thus, these responses to drought stress represented similar strategies. From the aspect of aboveground dry weight of plant without the ears (BIOM), GroupDs-3 remarkably exceeded (108.99%) the change% average of 28 barleys, while BIOM of GroupDs-1 and 2 remained well below it (77.16 and 70.26%, respectively).

### Genotypic differences in response to combined drought stresses

**Fig 4:**
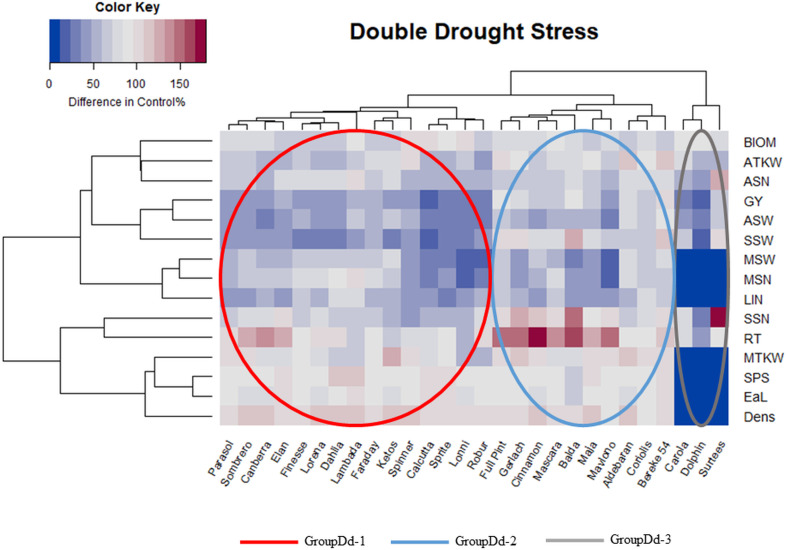
Heatmap combined with dendrograms of 28 barley genotypes (columns) and 15 traits (rows) using a combined drought stress at first node appearance (Z31) then at booting stage (Z49), expressed through changes in the percentage of the control values. The statistical methods used and the results visualized were the same as the ones used by our research group [77]. **Abbreviations**: BIOM* – aboveground dry weight of plant without the ears, ATKW – average thousand kernel weight, ASN – average number of grains per spikes harvested, GY – grain yield per plant, ASW – average weight of grains per spikes harvested, SSW – total grain weight in the side ears, MSW – weight of the grains in the main ear, MSN – number of grains in the main ear, LIN* – length of last internode, SSN – total grain number in side ears, RT – number of reproductive tillers, MTKW – thousand kernel weight in the main ear, SPS* – number of spikelets in the main ear, EaL* – length of the main ear, DENS* – main ear density. *morphological traits.

**Fig 5:**
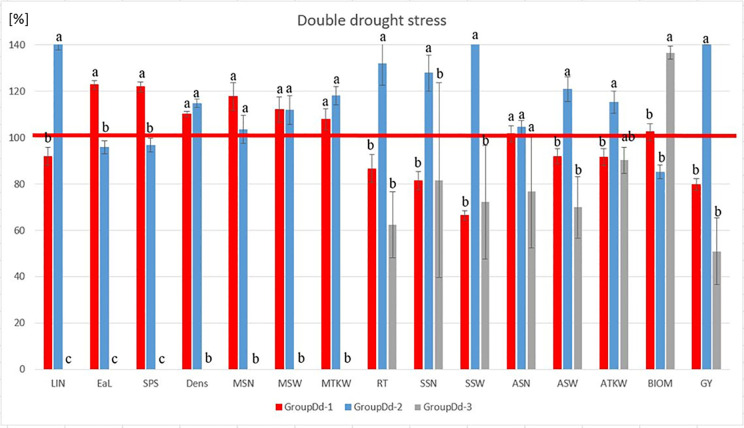
Evaluation of the stress responses of the three barley groups (GroupDd), identified in the heatmap using a combined drought stress treatment at first node appearance (Z31) and booting stage (Z49). (The calculation of group averages is based on the individual genotypic change percentage values, expressed as a proportion of the overall average change percentage of the 28 genotypes. The bars represent the standard deviation of the group members for each parameter. The same letters represented are not significantly different from one another at the P = 0.05 level). The statistical methods used and the results visualized were the same as the ones used by our research group before [77]. **Abbreviations**: LIN* – length of last internode, EaL* – length of the main ear, SPS* – number of spikelets in the main ear, DENS* – main ear density, MSN – number of grains in the main ear, MSW – weight of the grains in the main ear, MTKW – thousand kernel weight in the main ear, RT – number of reproductive tillers, SSN – total grain number in side ears, SSW – total grain weight in the side ears, ASN – average number of grains per spikes harvested, ASW – average weight of grains per spikes harvested, ATKW – average thousand kernel weight, BIOM* – aboveground dry weight of plant without the ears, GY – grain yield per plant. *morphological traits.

In the combined drought stress treatment, the correlations between the different traits followed a similar pattern as in the single drought stress treatment, with one major exception (Fig 4). Whereas in the single drought stress, the number of reproductive tillers (RT) was entirely separated from all the other traits, here it was placed nearest to the total grain number in the side ears (SSN). Interestingly, fewer genotypes were seriously affected by the combined drought stress. Based on the results of the combined drought stress, the 28 barley genotypes were grouped in three subclusters (GroupDd). The ratio of positive changes in the morphological and in most of the yield related traits, which was characteristic the GroupDs-1 and GroupDs-2 under the single drought, was similar (Fig 4). Genotypes in the GroupDd-1 and GroupDd-2 were the most efficient in maintaining MSN and MSW during the stress (Fig 5). They were also better at conserving RT, SSN and SSW Genotypes of GroupDd-3 showed a pattern similar to that in the single drought stress. The aboveground dry weight of plant without the ears (BIOM) of GroupDd-3 remarkably exceeded (107.83%) the change% average of 28 barleys, while BIOM of GroupDd-1 and 2 remained well below it (80.23 and 67.24%, respectively). The parameters of the main ear were almost zero, and the total grain yield could not be compensated by the side ears parameters (Fig 5). In summary, cultivars of GroupDd-1 and especially GroupDd-2 represented the more drought stress tolerant genotypes, possessing better recovering and compensating capacities after the stress ceased. This strategy has also been shown to be effective for better preservation of GY under combined stress.

There was a pronounced genotypic overlap between the similar reaction type groups (Group-1 and Group-2) identified in the two stress treatments (S1 Table). Six cultivars of the stress tolerant GroupDs-2 from the single drought stress formed the moderate stress tolerant GroupDd-1 of the combined drought stress. These six cultivars were ʻDahliaʼ, ʻElanʼ, ʻFaradayʼ, ʻParasolʼ, ʻRoburʼ and ʻSombreroʼ (S1 Table). In addition, eight of the 14 barley varieties identified in the single drought stress treatment showing better compensating ability (GroupDs-2) were able to maintain this ability under combined drought stress conditions as well (GroupDd-2); these were ʻAldebaranʼ, ʻBaldaʼ, ʻBereke 54ʼ, ʻCinnamonʼ, ʻCoriolisʼ, ʻFull Pintʼ, ʻMajaʼ and ʻMascaraʼ (S1 Table). The least drought tolerant GroupDs-3 and GroupDd-3 contained ʻCarolaʼ, ʻDolphinʼ and ʻSurteesʼ in both cases. ʻLambadaʼ, ʻSpinnerʼ, ʻKetosʼ and ʻLonniʼ (members of GroupDs-3), on the other hand, have retrieved the moderate ability due to repeated exposure to drought and have become members of the moderate tolerant group (GroupDd-1).

**Fig 6:**
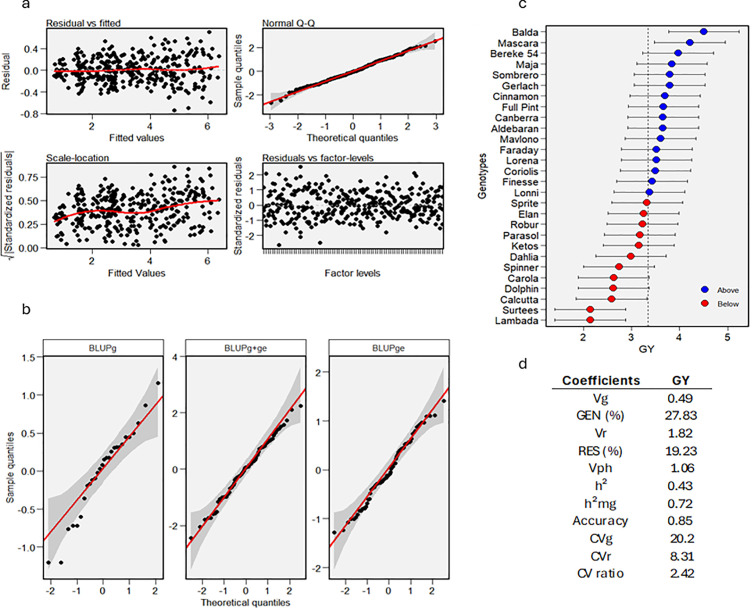
A linear mixed-effects model was used to (a) generate diagnostic plots of residuals, (b) visualize the normality of the random effects of genotype and interaction effects, (c) plot the Best Linear Unbiased Predictions (BLUPs) for predicted grain yield (GY), and (d) estimate the variance components and genetic coefficients for GY in 28 barley genotypes. Blue and red circles represent the genotypes that had BLUP above and below of BLUP means, respectively. Horizontal error bars represent the 95% confidence interval of prediction considering a two-tailed t test [92]. Abbreviations: g: genetypic values; ge: genotype × environment interaction; Vg: genotypic variance; Gen (%): genotypic variance percentage; Vr: residual variance; Res (%): residual variance percentage; Vph: phenotypic variance; h²: heritability of individual plots in the broad sense; h²mg: heritability of genotype mean, assuming complete survival; Accuracy: selective accuracy assuming no loss of plots; CVg: genotypic coefficient of variation; CVr: residual coefficient of variation; GY: Grain Yield.

Analyses of drought tolerance/susceptibility indices (DSI, STI, YSI, and TOL) revealed, that the genotypes ʻBereke 54ʼ, ʻBaldaʼ, ʻCinnamonʼ, and ʻFull Pintʼ (where DSI < 0.4; YSI ≥ 0.75; STI ≥ 1 and TOL value was the lowest) were the most drought-tolerant across all four indices. In contrast, the genotypes ʻDolphinʼ, ʻCarolaʼ, ʻRoburʼ, ʻLambadaʼ, ʻSurteesʼ, and ʻCalcuttaʼ (where DSI > 1.5; YSI < 0.3; STI < 0.2 and TOL value was the highest) were identified as the most susceptible to drought stress (S2 Fig). These findings are consistent with the results obtained from the BLUP-based analysis. The BLUP of mean grain yield rankings and the predicted mean values for grain yield (GY) across 28 barley genotypes is presented in Fig 6. Based on the results, ʻBaldaʼ, ʻMascaraʼ, and ʻBereke 54ʼ were identified as highly stable and high-yielding genotypes. In contrast, ʻLambadaʼ and ʻSurteesʼ were found to be unstable and low-yielding (Fig 6).

### Interaction system of morphological and yield related traits depending on the treatments

**Fig 7:**
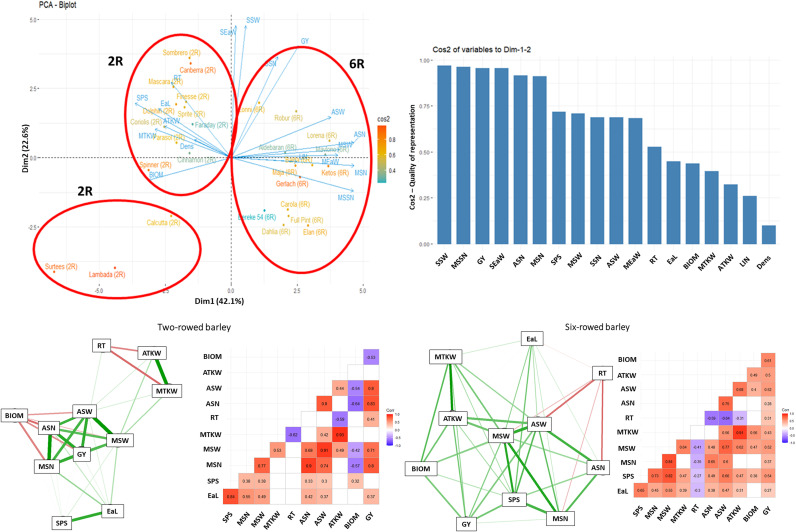
Principal Component Analysis (PCA) of trait and genotype biplots, along with trait-factor correlations and interaction systems, were performed on the average data matrices of morphological and yield-related traits from 28 barley genotypes, during which their control treatment measured under controlled environmental conditions were compared. 2R – two rowed, 6R – six rowed. **Abbreviations**: SSW – total grain weight in the side ears, MSSN – seed number per spikelet of the main ear, GY – grain yield per plant, SEaW – total side ears weight, ASN – average number of grains per spikes harvested, MSN – number of grains in the main ear, SPS* – number of spikelets in the main ear, MSW – weight of the grains in the main ear, SSN – total grain number in side ears, ASW – average weight of grains per spikes harvested, MEaW – main ear weight, RT – number of reproductive tillers, EaL* – length of the main ear, BIOM* – aboveground dry weight of plant without the ears, MTKW – thousand kernel weight in the main ear, ATKW – average thousand kernel weight, LIN* – length of last internode, DENS* – main ear density. *morphological traits.

**Fig 8:**
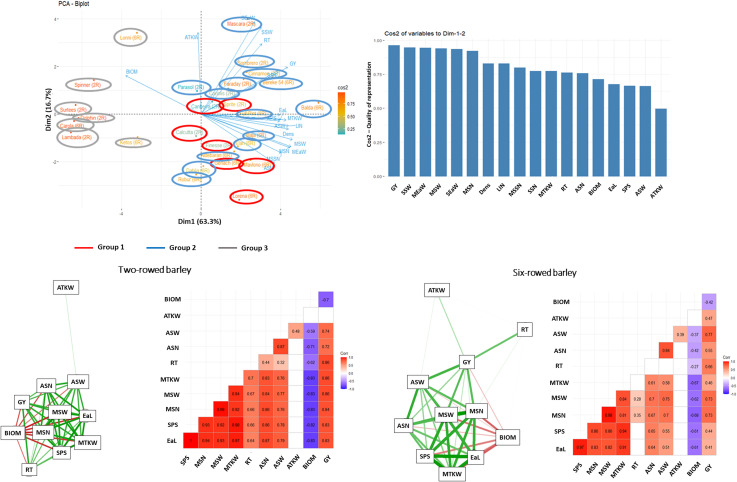
Principal Component Analysis (PCA) of trait and genotype biplots, along with trait-factor correlations and interaction systems, were performed on the average data matrices of morphological and yield-related traits from 28 barley genotypes, during which their single drought stress responses measured under controlled environmental conditions were compared. **Abbreviations**: GY – grain yield per plant, SSW – total grain weight in the side ears, MEaW – main ear weight, MSW – weight of the grains in the main ear, SEaW – total side ears weight, MSN – number of grains in the main ear, DENS* – main ear density, LIN* – length of last internode, MSSN – seed number per spikelet of the main ear, SSN – total grain number in side ears, MTKW – thousand kernel weight in the main ear, RT – number of reproductive tillers, ASN – average number of grains per spikes harvested, BIOM* – aboveground dry weight of plant without the ears, EaL* – length of the main ear, SPS* – number of spikelets in the main ear, ASW – average weight of grains per spikes harvested, ATKW – average thousand kernel weight. *morphological traits.

**Fig 9:**
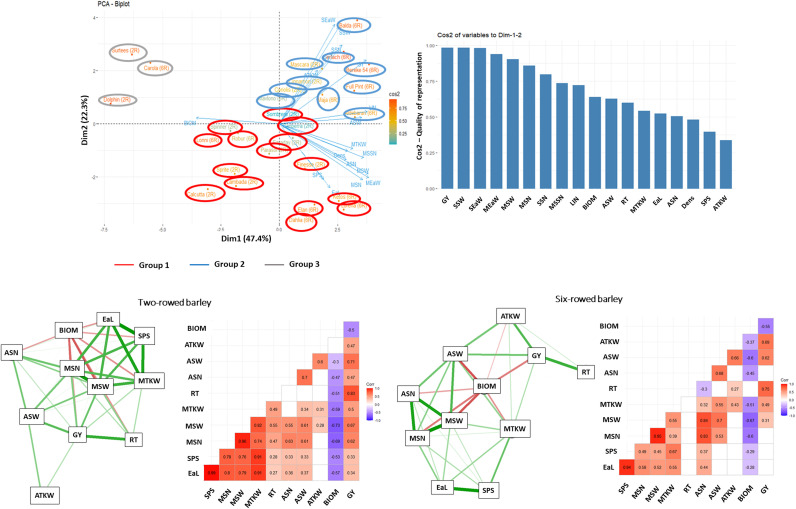
Principal Component Analysis (PCA) of trait and genotype biplots, along with trait-factor correlations and interaction systems, were performed on the average data matrices of morphological and yield-related traits from 28 barley genotypes, during which their double drought stress responses measured under controlled environmental conditions were compared. **Abbreviations**: GY – grain yield per plant, SSW – total grain weight in the side ears, SEaW – total side ears weight, MEaW – main ear weight, MSW – weight of the grains in the main ear, MSN – number of grains in the main ear, SSN – total grain number in side ears, MSSN – seed number per spikelet of the main ear, LIN* – length of last internode, BIOM* – aboveground dry weight of plant without the ears, ASW – average weight of grains per spikes harvested, RT – number of reproductive tillers, MTKW – thousand kernel weight in the main ear, EaL* – length of the main ear, ASN – average number of grains per spikes harvested, DENS* – main ear density, SPS* – number of spikelets in the main ear, ATKW – average thousand kernel weight. *morphological traits.

The network system of morphological and yield related traits studied was significantly altered both by the treatments and the row-type. For the control, the number of reproductive tillers (RT) in two-rowed barley showed a strong negative relationship with the thousand kernel weight traits (ATKW and MTKW), while in six-rowed barley, RT showed a similar relationship with the average number and average weight of grains per spike (ASN and ASW). Furthermore, a strong positive relationship was observed between the number and weight of grains for both row types, which showed a negative correlation with BIOM. In the case of six-rowed type, this negative relationship was not observed. Under the stress treatments (both single and double drought), however BIOM showed a strong negative relationship with the grain number and weight traits for both row-types (Fig 7–9).

In each treatment, the correlations between the morphological and grain yield related traits tested across the 28 barley cultivars were also evaluated by principal component analysis. The results of the biplot analysis were in good agreement with the heatmap dendrograms. The Eigenvalue of the first four factors was higher than 1, explaining together 89.7% of the phenotypic variance. In single stress treatment, the first and the second component explained 80% of the phenotypic variance (Fig 8), whereas in the double stress treatment their combined value was 69.7% (Fig 9). In the control treatment, the cultivars were basically separated based on the row type, with an additional separate group including three two-rowed genotypes (ʻSurteesʼ, ʻLambadaʼ and ʻCalcuttaʼ) (Fig 7). However, in both stress treatments (single and double drought) a clear separation of susceptible cultivars from drought tolerant and moderate tolerant genotypes could be observed (Fig 8,9).

## Discussion

Drought is one of the most important abiotic stressors, and its negative impact on plant production may become even more pronounced as a result of global climate change. Hence, it is a priority to study in details the genetic components of drought stress tolerance in economically important cereals, including barley. Drought stress response of plants highly depends on several parameters including the genotype. Its investigation is hindered by the often-observed low heritability under drought conditions which is frequently accompanied with high variance in genotype × environmental interactions [44,94]. To elucidate better the drought stress responses of different winter barley genotypes, our main aim was to compare the reaction of different barley cultivars to a single and to a combined drought stress treatment in controlled environmental conditions (growth chambers) on the morphological and grain yield components. For this purpose, we selected 28 barley cultivars based on the results of previous experiments [77,78]. Single drought stress was applied at the booting stage (Z49) and combined drought stress was applied at first node appearance and after a recovery period again at booting stage (Z31 + Z49). In the controlled experiment, we took advantage of the possibility to apply the stress at the same specific developmental phase for each genotype thus contributing to a more accurate evaluation of the genotypic stress responses.

The results of our experiment correspond well with already published data that most of the morphological and grain yield related traits were significantly affected by the drought stress [95–99]. The effect of drought stress during booting (Z49), and also the double stress treatment (Z31 + Z49), significantly decreased the grain number and weight components; a reduction of 35.91, 32.83 and 54.34, 51.89% of the control treatment was observed in single and combined drought stress, respectively. These results are consistent with previous studies [53,67,96,98,99]. In addition, our data also confirm that there is no significant difference in grain yield loss between barley and related species (e.g., wheat) under drought stress condition [100–103]. Water deficit also changed the relationships between the examined parameters, however the strong significant positive correlation between the main ear grain number and weight and the average grain number and weight were independent from treatment and the row-type. In addition, in both stress treatments (single and double drought) and for both row types, BIOM showed a strong negative relationship with the grain number and weight traits. This phenomenon is particularly well observed in the case of Group-3. Both in single and double drought stress treatments, the average value of BIOM in Group-3 remarkably exceeded the change% average of 28 barleys, while BIOM of GroupDd-1 and 2 remained well below it. However, genotypes in Group-1 and Group-2 were able to better retain the grain number and weight in the main ears (MSN, MSW and MTKW) during the drought stress period. In addition to the almost similar number of reproductive tillers, genotypes in Group-2 were able to produce a larger total grain number (SSN) and weights (SSW) during the recovery period in both stress treatments. It seems that the genotypes of Group-3 were not able to allocate resources into the grain yield of the main ears, instead, their vegetative mass was increased during the recovery period.

Based on the drought stress responses, the plants can be grouped into three main survival strategies, avoidance, tolerance and recovery [4]. The avoidance strategy involves maintaining a high-water potential in the plant by reducing stomatal transpiration losses [4,104–106]. They are also characterised by reduced plant productivity and reduced average size of vegetative and reproductive parts of the plant [4,83]. In this research, this type of drought stress mechanism was not possible to measure as the stress was applied in a developmental phase specific manner due to the controlled environmental conditions thus excluding stress avoidance completely. In addition, the other two types (tolerance and recovery) could not be clearly separated. The two groups of barley cultivars with positive drought stress responses could be characterised either with the ability of preserving grain number and weight in the main ears, in parallel of maintaining the number of reproductive tillers (more tolerant), or with the ability of preserving grain number and weight in the side ears (least tolerant).

We have confirmed that the hierarchical cluster analysis was successfully employed to determine the main stress response types among the barley varieties studied. In addition, by using drought tolerance/susceptibility indices (DSI, STI, YSI, and TOL) along with BLUP-based analysis, we identified genotypes, that exhibited the least grain yield loss under stress conditions. These cultivars demonstrated good yield stability and thus they represent promising candidates for breeding programs aimed at improving drought stress resistance. Separately, these analyses can reliably distinguish between drought-stress-tolerant and drought-stress-sensitive genotypes [31,107,108]. However, when used in combination, they enable more accurate selection in the development of stress-tolerant varieties [19,30].

In addition to the single drought stress, the application of double stress was also to serve to examine the possibility of stress priming in the case of drought. In most of the traits, the average values achieved in the combined stress were similar to those in the single stress or even lower, with the exception of the main ear density (Dens) and number of the reproductive tillers (RT) (Fig 1). Averaged over the 28 barley genotypes, the grain yield in the combined drought stress was 98.5% of that in the single drought stress with an interval of 43.3 and 190.1% (S1 Table). Fourteen cultivars of the 28 produced higher grain yield as in the single stress, though none was close to the control yield, and that was independent of the row-type. The drought stress priming seems to have a strong genotypic dependence as well, and it is also possible that the priming effect is more stress specific [109–111]. In addition, the duration of the somatic memory may be also different between the various stresses and genotypes [111]. The physiology and genetics behind these processes however are not well understood yet. When plants are exposed to drought stress, they undergo various physiological and biochemical changes, that enhance their drought tolerance [112]. These changes can be encoded at the epigenetic level and involve modifications such as DNA methylation, histone modification, and the expression of stress-responsive genes [113]. Drought can activate certain stress-responsive genes in the parent plants. Through the aforementioned epigenetic changes, these genes may also be activated in the offspring, resulting in a faster and more robust response to drought [114]. Our results, however underlined the existence of genetic diversity among barley genotypes in the extent of stress priming, that require further experiments to be clarified. Knowledge of stress memory may aid breeders in selecting parental plants with strong stress memory traits, which could lead to the development of more resilient barley varieties [115,116]. Therefore, our long-term goal is to investigate the epigenetic basis of drought stress-induced stress memory in barley in details, using a broader range of genetic material.

## Conclusion

We compared the drought stress responses for early and late developmental phases in various winter barley genotypes as measured under controlled environmental conditions. The main conclusion of this study is that, although both drought stress treatments (single and double) decreased the grain-yield related traits, there were no significant difference between them. Furthermore, detailed analysis of the data has identified two types of drought stress tolerance. In the combined drought stress experiment the phenomenon of drought priming could not be exactly clarified, but half of the cultivars showed an increase in the grain yield compared to the single stress. Most of the barley drought stress experiments focus on the terminal drought stress, our research demonstrated that the early water deficit during grain filling period or booting stage also can be decisive regarding the grain yield loss. Our results demonstrate diverse strategies of drought resistance in winter barley, with more drought-tolerant genotypes (e.g., GroupDd-2) offering valuable genetic resources for breeding programs aimed at mitigating yield loss amid the increasingly frequent and unpredictable drought events.

## Supporting information

S1 Fig The influence of genotype and drought stress (single and combined), expressed as the percentage of sum of squared variances (SS%), on different morphological (bold) and grain yield related traits within a cluster of 28 barley genotypes analyzed. The role of the drought stress treatment, the genotype as variant components in the yield components of the two rowed (a) and six rowed (b) barley varieties. *, **, *** denote significant relationships at the P ≤ 0.05, P ≤ 0.01 and P ≤ 0.001 probability levels, respectively; ns (not significant). The statistical methods used and the results visualized were the same as the ones used by our research group before [77].
(DOCX)

S1 Table The effect of drought stress on 28 barley genotypes in the controlled environmental drought stress experiment with their group positions and grain yields in single (Ds) and in double (Dd) drought stress treatments. The results were visualized in the same way as the ones used by our research group before [77].
(DOCX)

S2 Table Summary of 4-replicate data sets of control group genotypes and genotypes exposed to single and double drought stress conditions. (XLSX)

S2 Fig The Drought Susceptibility Index (DSI) (a), the Stress Tolerance Index (STI) (b), the Yield Stability Index (YSI), and the tolerance values were analyzed for grain yield (GY) of materials grown under control and drought conditions. Barley accessions with a DSI value ≤1 were considered more tolerant to drought stress (combined single and repeated treatments). GY_C: grain yield of control plants, GY (Drought): the average value of grain yield under drought stress conditions (single+double).
(DOCX)
