# Supplementary material for: Effect of drought stress during critical developmental stages on morphological and grain yield-related traits in winter barley (Hordeum vulgare L.)
Source: PLoS One. 2025 Jul 30;20(7):e0329391. doi: 10.1371/journal.pone.0329391 (PMC12309998; doi:10.1371/journal.pone.0329391)
Supplement: S1 Fig — The role of the drought stress treatment, the genotype as variant components in the yield components of the two rowed (a) and six rowed (b) barley varieties. *, **, *** denote significant relationships at the P ≤ 0.05, P ≤ 0.01 and P ≤ 0.001 probability levels, respectively; ns (not significant). The statistical methods used and the results visualized were the same as the ones used by our research group before [77]. (DOCX) [file pone.0329391.s001.docx]

**Supporting Information**

**effect of drought stress during Critical developmental stages on morphological and grain yield-related traits in winter Barley (*Hordeum vulgare* L.)**

Zita Berki^1^, Tibor Kiss^1,2*^, Judit Bányai^1^, András Cseh^1^, Krisztina Balla^1^, Ildikó Karsai^1,*^

^1^ *HUN-REN Centre for Agricultural Research, Agricultural Institute, H-2462 Martonvásár,* Hungary

^2^ *Food and Wine Research Institute, Eszterházy Károly Catholic University, H-3300 Eger, Hungary*

*Corresponding authors: kiss2.tibor@uni-eszterhazy.hu, karsai.ildiko@atk.hun-ren.hu

**S1 Fig** The influence of genotype and drought stress (single and combined), expressed as the percentage of sum of squared variances (SS%), on different morphological (bold) and grain yield related traits within a cluster of 28 barley genotypes analyzed. The role of the drought stress treatment, the genotype as variant components in the yield components of the two rowed (a) and six rowed (b) barley varieties. *, **, *** denote significant relationships at the P ≤ 0.05, P ≤ 0.01 and P ≤ 0.001 probability levels, respectively; ns (not significant). The statistical methods used and the results visualized were the same as the ones used by our research group before [77].


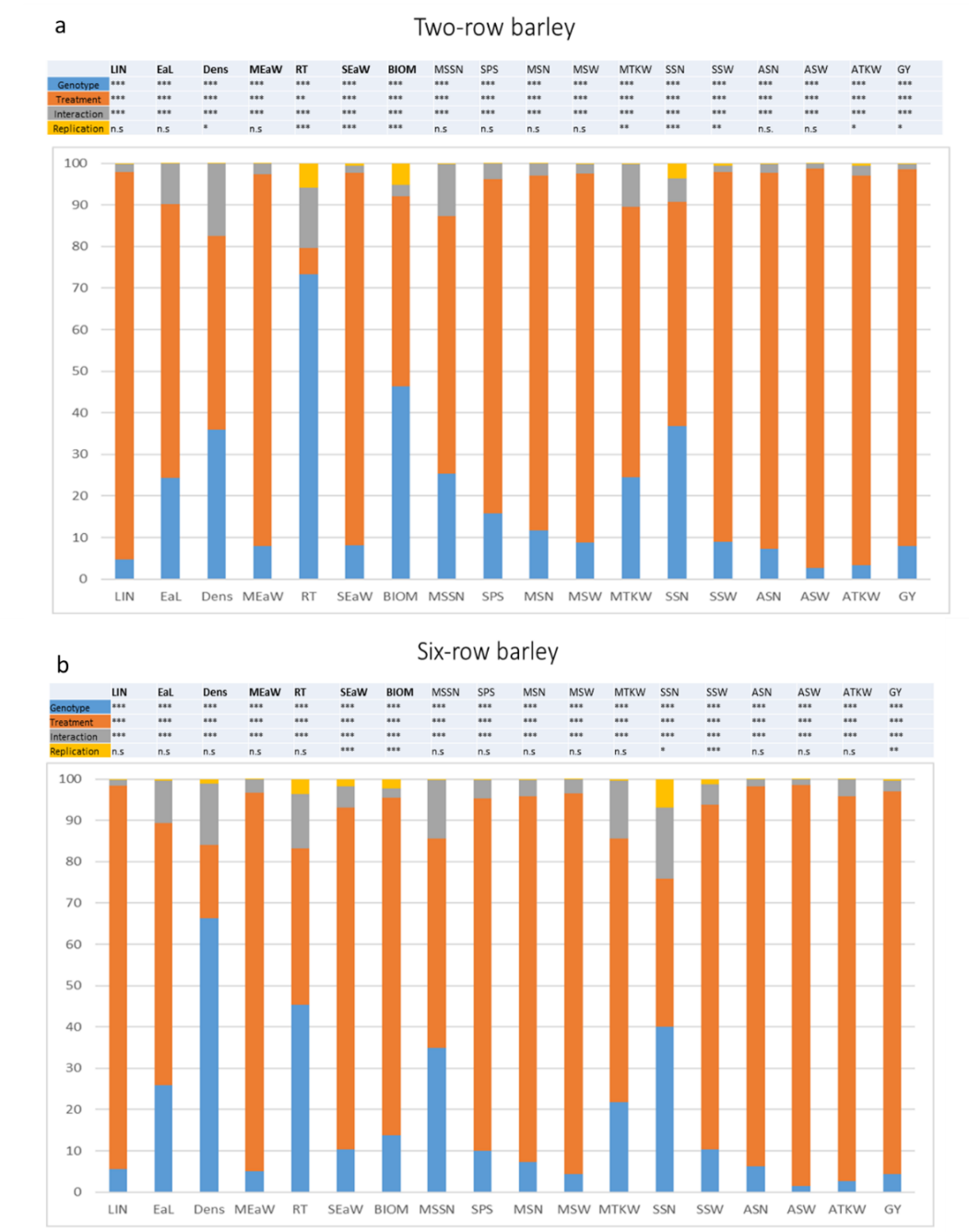


**Abbreviations**:

LIN* – length of last internode, EaL* – length of the main ear, DENS* – main ear density, MEaW – main ear weight, RT – number of reproductive tillers, SEaW – total side ears weight, BIOM* – aboveground dry weight of plant without the ears, MSSN – seed number per spikelet of the main ear, SPS* – number of spikelets in the main ear, MSN – number of grains in the main ear, MSW – weight of the grains in the main ear, MTKW – thousand kernel weight in the main ear, SSN – total grain number in side ears, SSW – total grain weight in the side ears, ASN – average number of grains per spikes harvested, ASW – average weight of grains per spikes harvested, ATKW – average thousand kernel weight, GY – grain yield per plant

*morphological traits
