# Supplementary material for: Effect of drought stress during critical developmental stages on morphological and grain yield-related traits in winter barley (Hordeum vulgare L.)
Source: PLoS One. 2025 Jul 30;20(7):e0329391. doi: 10.1371/journal.pone.0329391 (PMC12309998; doi:10.1371/journal.pone.0329391)
Supplement: S1 Table — The results were visualized in the same way as the ones used by our research group before [77]. (DOCX) [file pone.0329391.s002.docx]

**Supporting Information**

**effect of drought stress during Critical developmental stages on morphological and grain yield-related traits in winter Barley (*Hordeum vulgare* L.)**

Zita Berki^1^, Tibor Kiss^1,2*^, Judit Bányai^1^, András Cseh^1^, Krisztina Balla^1^, Ildikó Karsai^1,*^

^1^ *HUN-REN Centre for Agricultural Research, Agricultural Institute, H-2462 Martonvásár,* Hungary

^2^ *Food and Wine Research Institute, Eszterházy Károly Catholic University, H-3300 Eger, Hungary*

*Corresponding authors: kiss2.tibor@uni-eszterhazy.hu, karsai.ildiko@atk.hun-ren.hu

**S1 Table** The effect of drought stress on 28 barley genotypes in the controlled environmental drought stress experiment with their group positions and grain yields in single (Ds) and in double (Dd) drought stress treatments. The results were visualized in the same way as the ones used by our research group before [77].

| **Genotype** | **Spike Type** | **Single stress Group number** | **Combined stress Group number** | **Control Grain Yield [g]** | **Single stress Grain Yield [g]** | **Combined stress Grain Yield**  **[g]** | **Drought priming effect on Grain Yield**  **[Dd/Ds%]** |
| --- | --- | --- | --- | --- | --- | --- | --- |
| Calcutta | 2R | 1 | 1 | 3.86 | 2.09 | 0.91 | 43.3 |
| Canberra | 2R | 1 | 1 | 6.01 | 2.61 | 2.73 | 104.4 |
| Finesse | 2R | 1 | 1 | 5.57 | 2.38 | 2.43 | 102.4 |
| Gerlach | 6R | 1 | 2 | 5.12 | 2.65 | 4.14 | 156.4 |
| Lorena | 6R | 1 | 1 | 5.93 | 2.25 | 2.60 | 115.5 |
| Mavlono | 6R | 1 | 2 | 5.72 | 2.89 | 2.52 | 87.2 |
| Sprite | 2R | 1 | 1 | 5.46 | 2.98 | 1.51 | 50.6 |
| **Aldebaran** | **6R** | **2** | **2** | **4.99** | **2.72** | **3.63** | **133.7** |
| **Balda** | **6R** | **2** | **2** | **5.37** | **4.86** | **4.67** | **96** |
| **Bereke 54** | **6R** | **2** | **2** | **4.23** | **4.13** | **4.31** | **104.4** |
| **Cinnamon** | **2R** | **2** | **2** | **4.65** | **3.52** | **3.33** | **94.6** |
| **Coriolis** | **2R** | **2** | **2** | **4.67** | **2.87** | **3.14** | **109.8** |
| Dahlia | 6R | 2 | 1 | 4.64 | 1.93 | 1.96 | 101.6 |
| Elan | 6R | 2 | 1 | 4.53 | 2.86 | 2.24 | 78.4 |
| Faraday | 2R | 2 | 1 | 4.79 | 3.41 | 2.59 | 75.9 |
| **Full Pint** | **6R** | **2** | **2** | **4.30** | **3.31** | **3.77** | **113.8** |
| **Maja** | **6R** | **2** | **2** | **5.26** | **3.50** | **3.36** | **96.1** |
| **Mascara** | **2R** | **2** | **2** | **5.69** | **4.03** | **3.95** | **98** |
| Parasol | 2R | 2 | 1 | 4.71 | 2.43 | 2.18 | 89.6 |
| Robur | 6R | 2 | 1 | 6.27 | 1.67 | 1.60 | 95.8 |
| Sombrero | 2R | 2 | 1 | 5.69 | 3.62 | 2.62 | 72.4 |
| Carola | 6R | 3 | 3 | 4.68 | 0.81 | 1.54 | 190.1 |
| Dolphin | 2R | 3 | 3 | 5.21 | 1.03 | 0.77 | 75.1 |
| **Ketos** | **6R** | **3** | **1** | **5.20** | **1.56** | **2.47** | **157.8** |
| **Lambada** | **2R** | **3** | **1** | **2.86** | **0.76** | **1.38** | **182.2** |
| **Lonni** | **6R** | **3** | **1** | **5.96** | **2.74** | **1.45** | **52.7** |
| **Spinner** | **2R** | **3** | **1** | **4.24** | **1.56** | **1.71** | **109.3** |
| Surtees | 2R | 3 | 3 | 2.44 | 0.99 | 1.56 | 157.2 |
