# Supplementary material for: Effect of drought stress during critical developmental stages on morphological and grain yield-related traits in winter barley (Hordeum vulgare L.)
Source: PLoS One. 2025 Jul 30;20(7):e0329391. doi: 10.1371/journal.pone.0329391 (PMC12309998; doi:10.1371/journal.pone.0329391)

**Supporting Information**

**effect of drought stress during Critical developmental stages on morphological and grain yield-related traits in winter Barley (*Hordeum vulgare* L.)**

Zita Berki^1^, Tibor Kiss^1,2*^, Judit Bányai^1^, András Cseh^1^, Krisztina Balla^1^, Ildikó Karsai^1,*^

^1^ *HUN-REN Centre for Agricultural Research, Agricultural Institute, H-2462 Martonvásár,* Hungary

^2^ *Food and Wine Research Institute, Eszterházy Károly Catholic University, H-3300 Eger, Hungary*

*Corresponding authors: kiss2.tibor@uni-eszterhazy.hu, [karsai.ildiko@atk.hun-ren.hu](mailto:karsai.ildiko@atk.hun-ren.hu)

**S2 Fig** The Drought Susceptibility Index (DSI) (a), the Stress Tolerance Index (STI) (b), the Yield Stability Index (YSI), and the tolerance values were analyzed for grain yield (GY) of materials grown under control and drought conditions. Barley accessions with a DSI value ≤1 were considered more tolerant to drought stress (combined single and repeated treatments). GY_C: grain yield of control plants, GY (Drought): the average value of grain yield under drought stress conditions (single+double)


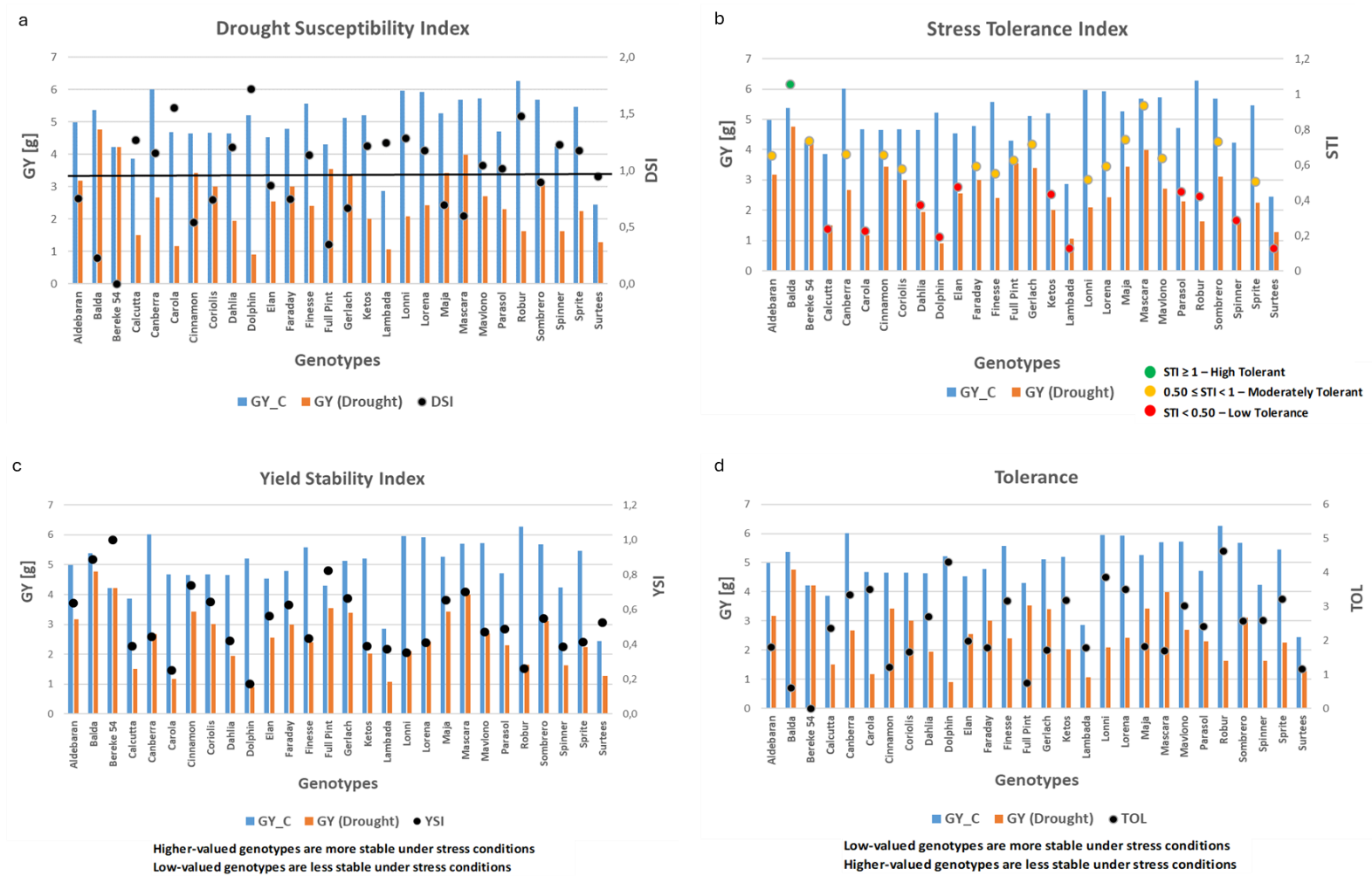

Supplement: S2 Fig — Barley accessions with a DSI value ≤1 were considered more tolerant to drought stress (combined single and repeated treatments). GY_C: grain yield of control plants, GY (Drought): the average value of grain yield under drought stress conditions (single+double). (DOCX) [file pone.0329391.s004.docx]
